# Microwave-Assisted Synthesis of Potential Bioactive Benzo-, Pyrido- or Pyrazino-thieno[3,2-*d*]pyrimidin-4-amine Analogs of MPC-6827

**DOI:** 10.3390/ph13090202

**Published:** 2020-08-19

**Authors:** Yvonnick Loidreau, Marie-Renée Nourrisson, Corinne Fruit, Cécile Corbière, Pascal Marchand, Thierry Besson

**Affiliations:** 1 Normandie Univ, UNIROUEN, INSA Rouen, CNRS, COBRA UMR 6014, 76000 Rouen, France; yvonnick.loidreau@gmail.com (Y.L.); corinne.fruit@univ-rouen.fr (C.F.); 2 Université de Nantes, Cibles et Médicaments des Infections et du Cancer, IICiMed, EA 1155, F-44000 Nantes, France; marie-renee.nourrisson@univ-nantes.fr; 3 Normandie Univ, UNIROUEN, ABTE, 76000 Rouen, France; cecile.corbiere@univ-rouen.fr

**Keywords:** microwave-assisted chemistry, thieno[3,2-*d*]pyrimidines, colorectal cancer, HT-29 cells, caco-2 cells, antiproliferative activity

## Abstract

Efficient microwave-assisted chemical processes were applied to the synthesis of an array of novel *N*-(4-methoxyphenylamino)-2-methyl benzo-, pyrido- or pyrazino-thieno[3,2-*d*]pyrimidin-4-amine derivatives. These heteroaromatic systems were envisioned as potent bioisosteric analogues of **MPC-6827**, an anticancer agent previously developed until phase II clinical studies. A brief evaluation and comparison of their antiproliferative activity on HT-29 and Caco-2, two human colorectal cancer cell lines, were also reported. At the tested concentrations (5 and 10 µM), thieno[3,2-*d*]pyrimidin-4-amines **4a** and **4c** exhibited an inhibitory effect similar to **MPC-6827** on human colorectal cancer cell proliferation.

## 1. Introduction

Due to their presence in numerous biologically active compounds, quinazoline derivatives are of particular interest for medicinal chemistry and remain a major research area in organic chemistry [1,2,3,4,5]. Among the numerous bioactive quinazolines, **MPC-6827** (*N*-(4-methoxyphenylamino)-*N*,2-dimethylquinazoline) has been extensively studied for its therapeutic use against cancer [6,7]. **MPC-6827**, also named Azixa or verubulin, is a microtubule-destabilizing agent exhibiting a dual mode of action, leading to apoptosis by blocking cell cycle and to growth inhibition on several types of cancer such as breast, colon and ovarian cancers [8,9,10]. **MPC-6827** is also known to reduce blood supply to the tumors [11]. Based on these data, this benzo[*e*]pyrimidine emerged as a good candidate for phase I and phase II clinical trials in patients with metastatic melanoma and glioblastoma multiforme [12,13,14]. Despite these investigations revealing some cardiotoxicity and leading to the suspension of clinical development in phase II [15], **MPC-6827** remains an excellent model for the design of potential cytotoxic agents [16].

**Scheme 1:**
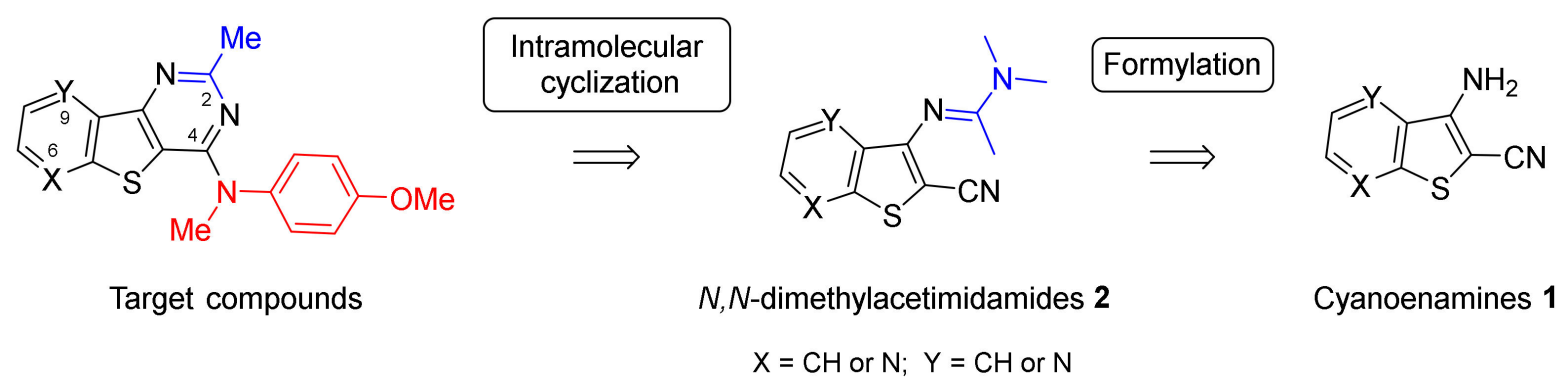
Proposed retrosynthetic route of the target tricyclic analogs of **MPC-6827**.

There are a few synthetic routes of **MPC-6827** reported in the literature. The initial work of Sirisoma and his co-workers was carried out in three steps from anthranilic acid methyl ester [9]. In the last step, 4-chloro-2-methylquinazoline was reacted with *N*-methyl-4-methoxyaniline in anhydrous propanol to give the target product in an overall yield of 55% (Scheme 1) [9]. These methods usually require forcing conditions with long reaction times and, sometimes, conditions using toxic reagents (e.g., POCl_3_).

**Figure 1:**
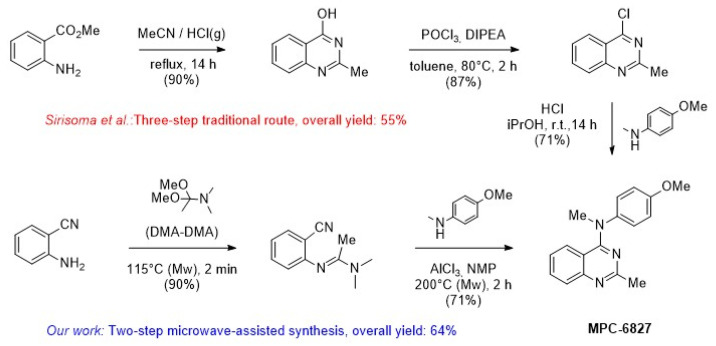
Synthetic routes of **MPC-6827** described by Sirisoma et al. and our group a decade ago.

To develop sustainable and convenient multicomponent processes for the synthesis of quinazoline and quinazolinone derivatives [17,18,19], our group investigated a novel and efficient two-step synthesis of **MPC-6827** [20]. Indeed, reaction of anthranilonitrile with *N*,*N*-dimethylacetamide dimethyl acetal (DMA-DMA) at 115 °C for 2 min gave acetimidamide intermediate in excellent yield (90%). It was intensely heated (200 °C for 2 h) with *N*-methyl-*p*-anisidine (1.5 equiv.), in *N*-methylpyrrolidone (NMP), in the presence of aluminium chloride (AlCl_3_, 1.5 equiv.). **MPC-6827** was obtained in 71% yield, i.e., 64% using the two-step synthesis method (Figure 1).

**Figure 2:**
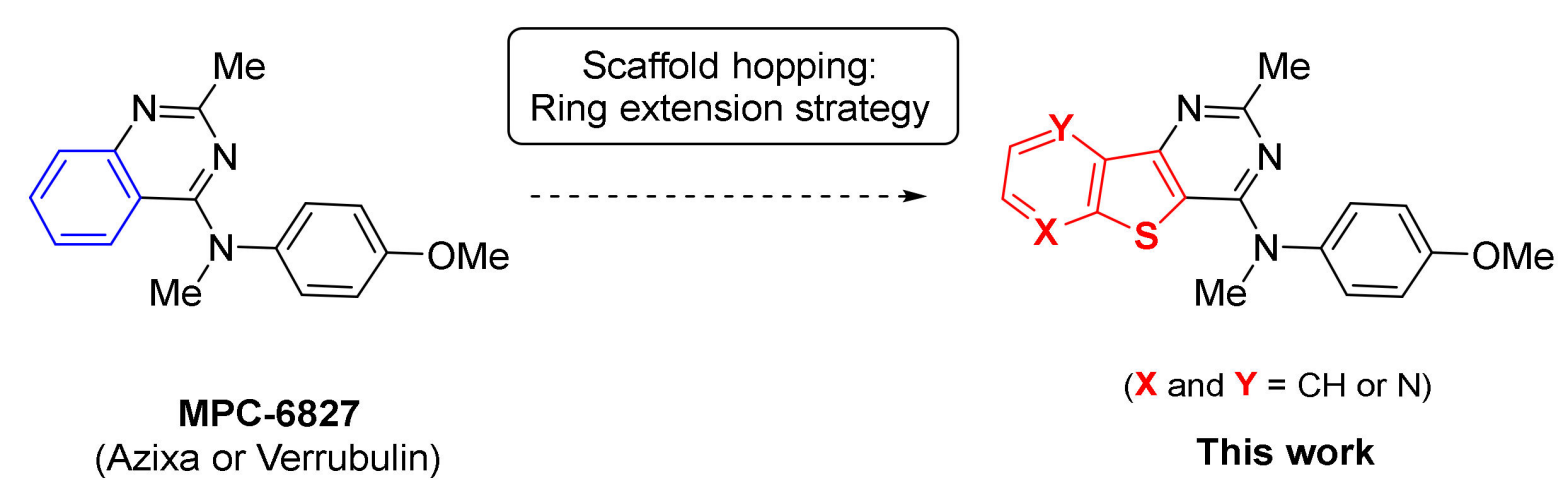
Structure of **MPC-6827** and *N*-(4-methoxyphenylamino)-2-methyl benzo-, pyrido- or pyrazino-thieno[3,2-*d*]pyrimidine derivatives envisioned in this work.

Combining our chemistry work with a scaffold hopping strategy, we envisioned to extend and replace the benzenic part of this small molecule into an aryl thiophene ring. Herein, we report the convenient synthesis of an array of novel *N*-(4-methoxyphenylamino)-2-methyl benzo-, pyrido- or pyrazino-thieno[3,2-*d*]pyrimidine derivatives, envisioned as bioisosteric analogs of **MPC-6827** (Figure 2). A brief evaluation of their antiproliferative activity on two human colorectal cancer cell lines (human HT-29 and Caco-2) is also reported and compared with data obtained for the source of inspiration (**MPC-6827**).

## 2. Results and Discussion

### 2.1. Chemistry

The target compounds were *N*-(4-methoxyphenyl)-*N*,2-dimethylthieno[3,2-*d*]pyrimidin-4-amine derivatives bearing *N*-methyl-*p*-anisidine at C4 position of the pyrimidinyl ring. As depicted in Scheme 1, the retrosynthetic route to the novel heteroarenes was envisioned via intramolecular cyclization of the key *N*,*N*-dimethylacetimidamides (**2**), obtained by formylation of the corresponding cyanoenamines (**1**).

**Scheme 2:**
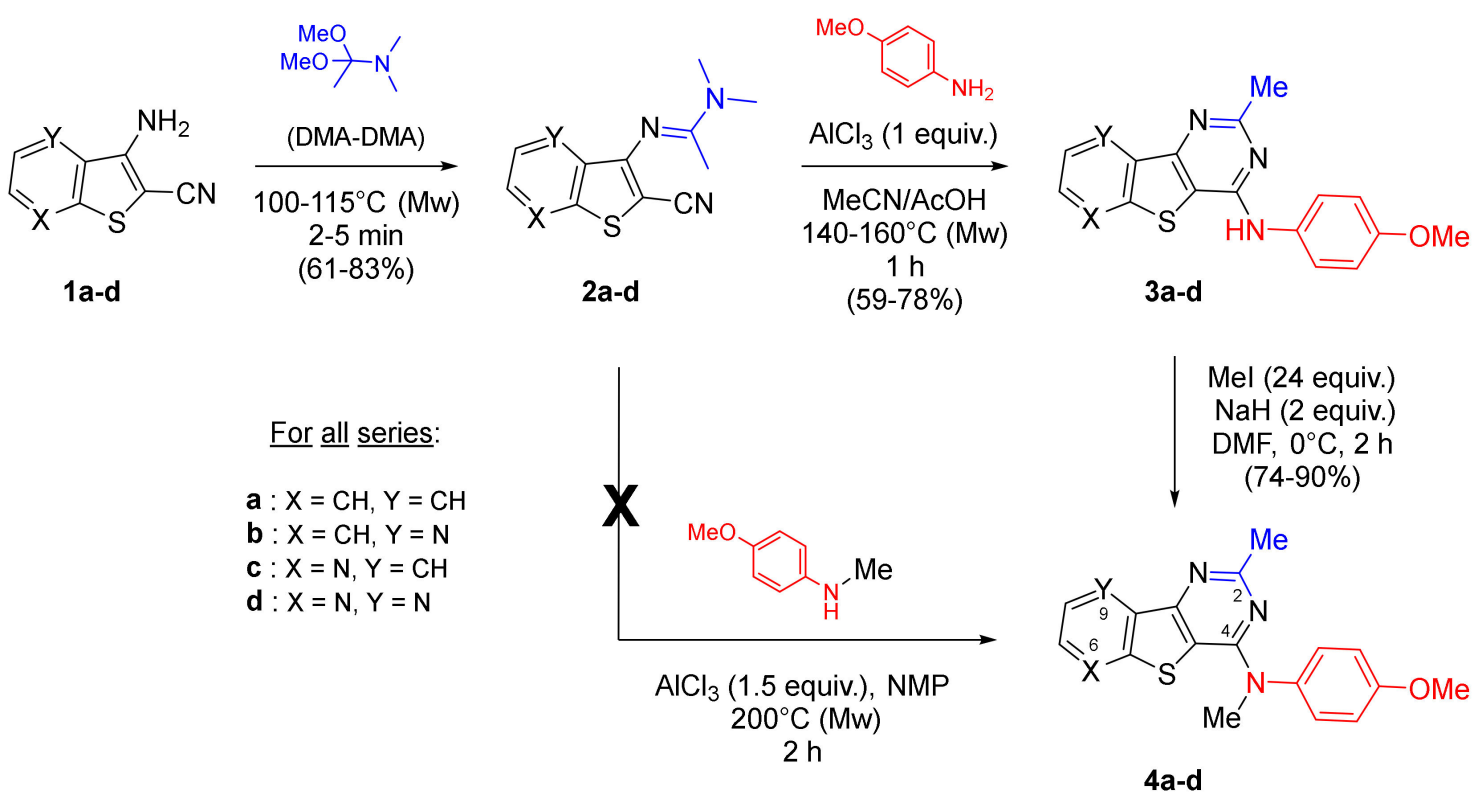
Synthesis of compounds **2**, **3** and **4** (see Table 1 for experimental conditions).

The first step of the process concerned the synthesis of *N*,*N*-dimethylacetimidamides (**2a**–**d**), which were obtained in good yields (61–83%) by heating the corresponding cyanoenamines (**1a**–**d**) with a large excess (10 equiv.) of DMA-DMA at 110–115 °C within 2–5 min of irradiation (Scheme 2).

According to our preceding work (Figure 1) [20], *(E)-N′*-(2-cyanobenzo[*b*]thiophen-3-yl)-*N*,*N*-dimethylacetimidamide (**2a**) was treated with *N*-methyl-*p*-anisidine and 1.5 equiv. of AlCl_3_ in *N*-methylpyrrolidone (NMP), and was heated at 200 °C for 2 h under microwave irradiation (Scheme 2). These operating conditions allowed the synthesis of **MPC-6827**; however, we were unable to generate its thiophenic analogue (**4a**) or the corresponding enamine intermediate resulting from the attack of the aromatic secondary amine on the activated carbonitrile group (Scheme 2).

Thus, an alternative two-step procedure starting from acetimidamide **2a** was considered (Scheme 2). Starting acetimidamide (**2a**) was heated at 118 °C with *p*-anisidine (1 equiv.) in acetonitrile/acetic acid (2:1; *v*/*v*) as solvent. However, after 30 min or 1 h of irradiation (400 W), the formation of *N*-(4-methoxyphenyl)-2-methyl-benzo[4,5]thieno[3,2-*d*]pyrimidin-4-amine (**3a**) was not complete and reagents were recovered in the final mixture. The addition of AlCl_3_ (1 equiv.) and an increase of reaction temperature until 160 °C allowed access to the attempted cyclized compound (**3a**) in good yield (78%). Alkylation of the exocyclic amine was realized under usual conditions by stirring (**3a**) with a large excess of iodomethane (CH_3_I) in the presence of sodium hydride (NaH), at 0 °C for 2 h. The expected *N*-(4-methoxyphenyl)-*N*,2-dimethylbenzo[4,5]thieno[3,2-*d*]pyrimidin-4-amine (**4a**) was then obtained in very good yield (86%) (Scheme 2, Table 1). This two-step sequence was applied to *N′*-(2-cyanothieno[3,2-*b*]pyridin-3-yl)-, *N′*-(2-cyanothieno[2,3-*b*]pyridin-3-yl)- and *N′*-(6-cyanothieno[2,3-*b*]pyrazin-7-yl)-*N,N*-dimethylacetimidamides (**2b**, **2c** and **2d**, respectively) to yield the final products **4b**, **4c** and **4d**, respectively (Scheme 2, Table 1).

Microwave-assisted heating is an efficient technology that allows reproducible and safe operating conditions for convenient access to various molecules when traditional multistep processes would need long reaction times and unstable and toxic reagents (e.g., formamide and POCl_3_) [21,22,23,24]. The innovative conditions previously described for the synthesis of **MPC-6827** failed to provide compounds **4a–d** and a more traditional approach was investigated.

**Scheme 3:**
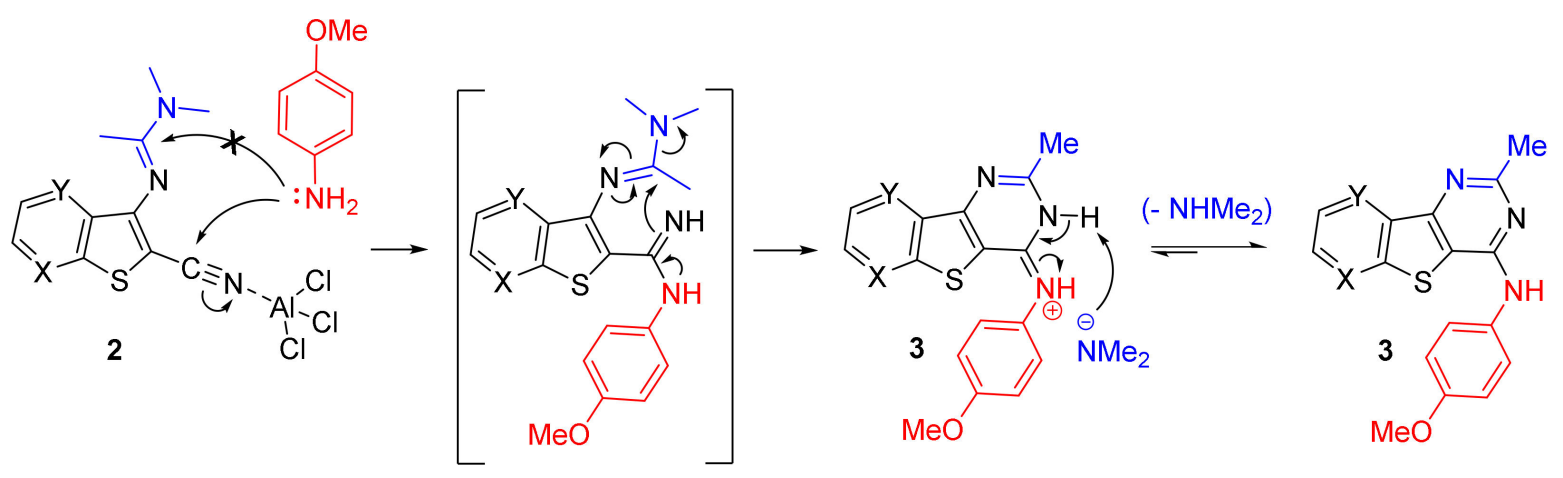
Suggested mechanism for reaction of **2** series with *p*-anisidine and access to **3**, then **4**.

The crucial part of our synthetic pathway was the cyclization step in which cyanoenamines **2** were converted into tricyclic compounds **3**. Despite our efforts, the synthesis of thienopyridines **3b** and **3c** remained difficult, as demonstrated by the low yields described in Table 1. The suggested mechanism is described in Scheme 3.

Based on the electrophilic character of the cyano group, the reaction starts by nucleophilic attack of the primary aromatic amine on the carbon of the cyano group, which is activated by AlCl_3_ [25]. The intermediate *p*-methoxyphenylamidine can then undergo intramolecular cyclization via the attack of the more nucleophilic amidino secondary amine to enamine function, generating the pyrimidine ring present in the expected *N*-(4-methoxyphenyl)-2-methyl-benzo[4,5]thieno[3,2-*d*]pyrimidin-4-amines (**3**). Interestingly, the release of the dimethylamino group, during the cyclisation step, would favor afterwards the aromatization step.

### 2.2. Antiproliferative Activity on Colorectal Cancer Cell Lines (Caco-2 and HT-29)

**Figure 3:**
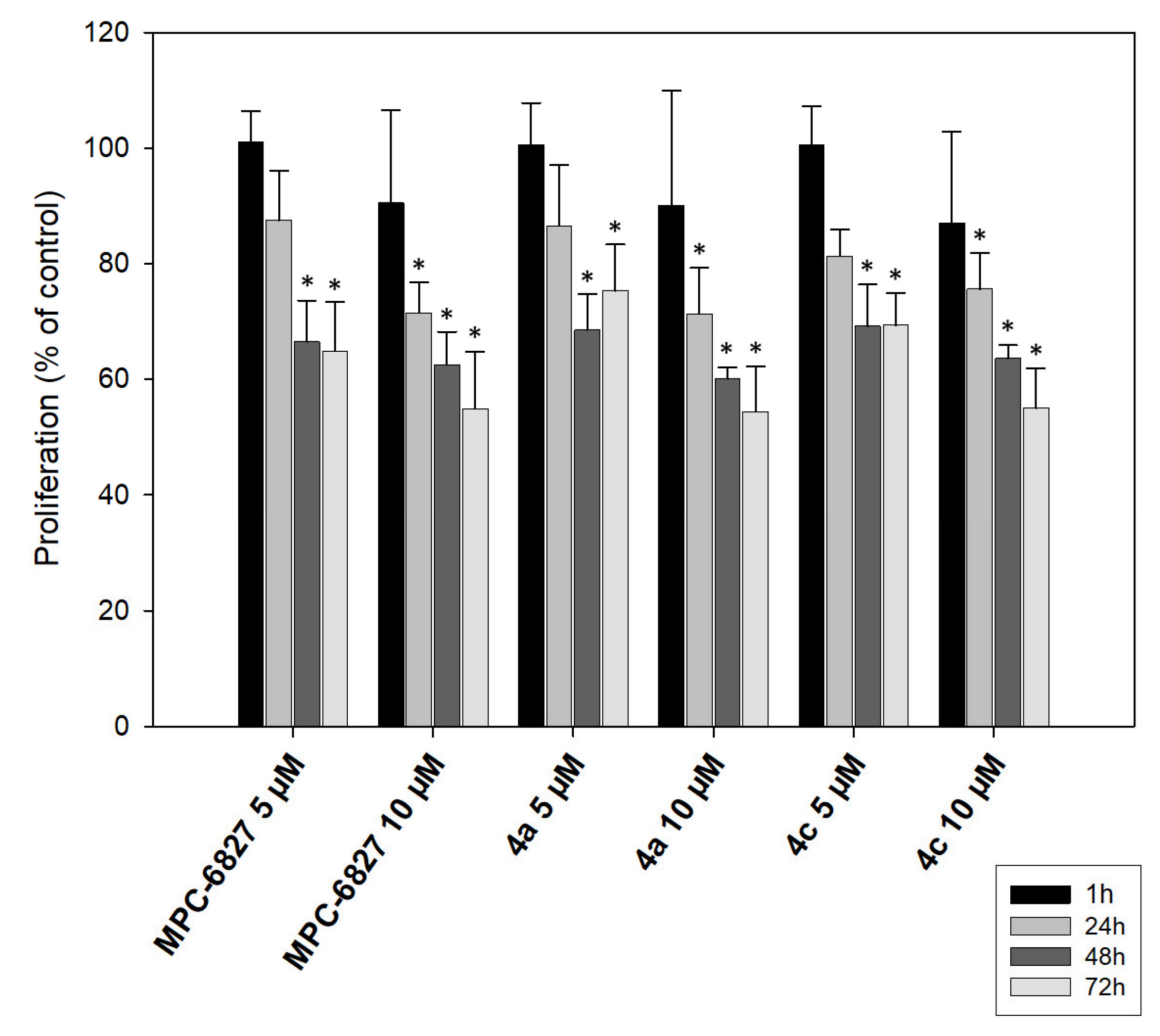
Proliferation of Caco-2 cells treated with **MPC-6827** (5 and 10 µM), **4a** (5 and 10 µM) and **4c** (5 and 10 µM) after 1, 24, 48 and 72 h of treatment. Proliferation was evaluated with an MTT assay. Data are reported as the mean ± SD in percentage from three independent experiments. * *P* < 0.05 vs. control that represented 100% of proliferation.

**Figure 4:**
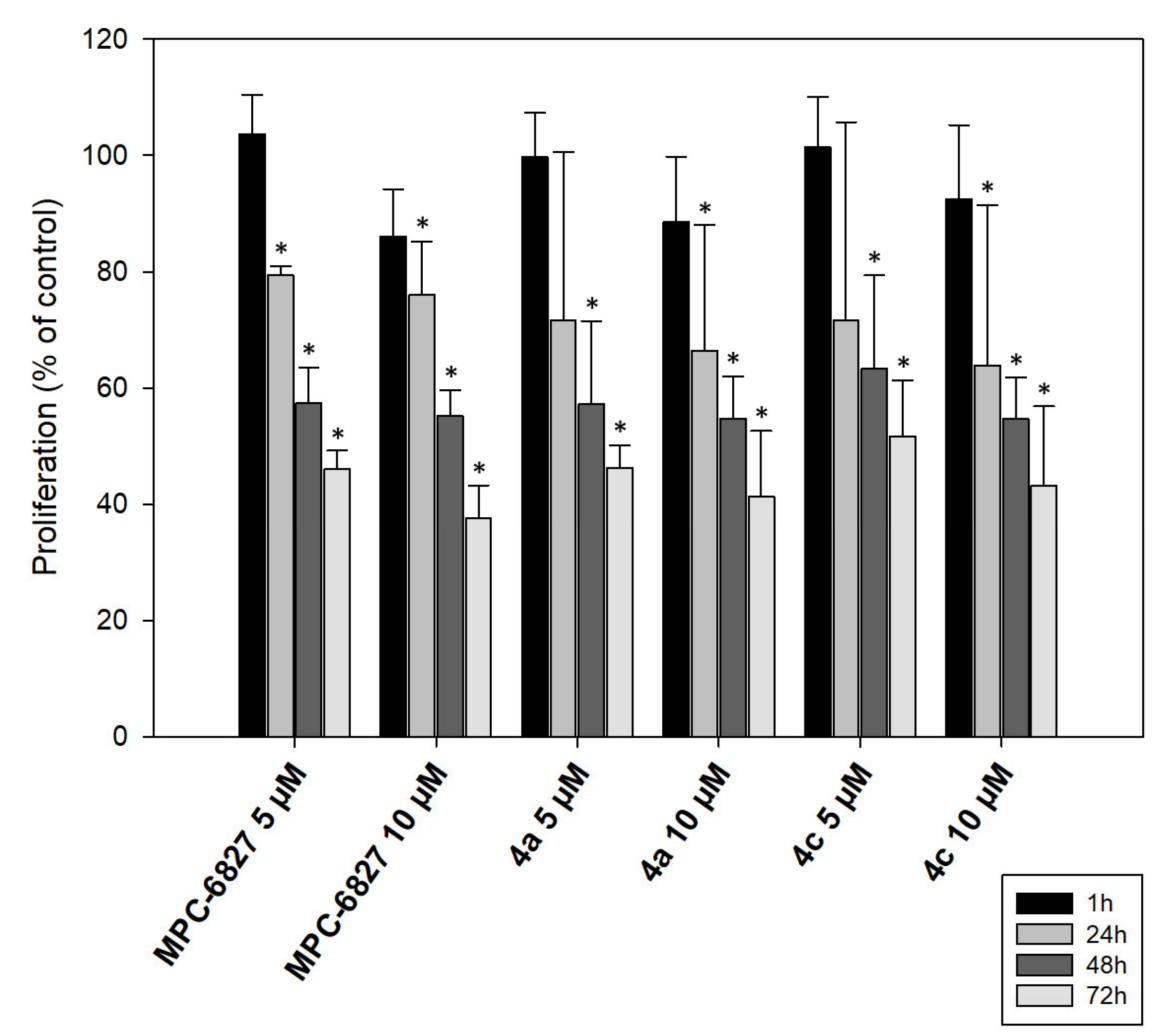
Proliferation of HT-29 cells treated with **MPC-6827** (5 and 10 µM), **4a** (5 and 10 µM) and **4c** (5 and 10 µM) after 1, 24, 48 and 72 h of treatment. Proliferation was evaluated with an MTT assay. Data are reported as the mean ± SD in percentage from three independent experiments. * *P* < 0.05 vs. control that represented 100% of proliferation.

In preliminary experiments, the antiproliferative effect of compounds (**3b–d**) and (**4a–d**) was evaluated on Caco-2 cells and compared with that of **MPC-6827**. Each molecule was tested at two concentrations (5 and 10 µM) for 1, 24, 48 and 72 h. The benzo[4,5]thieno[3,2-*d*]pyrimidine **3a** was not tested because of its insolubility in the conditions used. The proliferation of Caco-2 cells was not altered with molecules **3b**, **3c**, **3d**, **4b** and **4d** (data not shown). The two most interesting compounds (**4a** and **4c**) were also tested on HT-29 cells. Results obtained on Caco-2 and HT-29 cells for **4a**, **4c** and **MPC-6827** are presented in Figure 3 and Figure 4, respectively.

The results of the biological evaluation highlighted that the two compounds (**4a** and **4c**) that possessed antiproliferative properties on Caco-2 and HT-29 were as interesting as **MPC-6827** (Azixa or verubulin).

Overall, the number of surviving Caco-2 cancer cells decreased in a time-dependent manner. More precisely, inhibition of the proliferation of Caco-2 colon cancer cells was 25% after 24 h of treatment with 10 µM concentrations of **4c**. Higher inhibitory effects measured after 48 h of incubation were close to the final results observed after 72 h of experiment. In this case, proliferation of Caco-2 cells was significantly inhibited in the presence of **4a** and **4c**, in a manner equivalent to that of **MPC-6827**, with inhibitory effects of 30% and 45% at 5 µM and 10 µM, respectively (Figure 3).

Similar to Caco-2 cells, HT-29 cancer cell proliferation was significantly inhibited in the presence of **MPC-6827** and compounds **4a** and **4c**, after 72 h of treatment (Figure 4). The growth inhibition of the HT-29 colorectal cancer cell line also appeared to be time-dependent. In addition, more important effects were observed compared to those described for Caco-2 cells (around 50% and 60% inhibition at 5 µM and 10 µM concentration, respectively) (Figure 3 and Figure 4).

## 3. Conclusions

This work demonstrated that the novel thieno[3,2-*d*]pyrimidin-4-amines **4a** and **4c** exhibited a similar inhibitory effect on colon cancer cell proliferation as **MPC-6827**. The exchange of the carbon at position 6 of **4a** by a nitrogen atom, as in **4c**, appeared to maintain the biological activity studied. Furthermore, a comparison of results described for **4a** and **4c** with those obtained for **4b** and **4d** suggested that modifying the atom at position 9 of the heteroaromatic scaffold strongly decreased the biological effect. These preliminary results encourage us to carry on the development of such compounds in the hope of identifying new leads.

## 4. Materials and Methods

### 4.1. Chemistry

#### 4.1.1. General Information

All reagents were purchased from commercial suppliers and were used without further purification. All reactions were monitored by thin-layer chromatography with aluminium plates (0.25 mm) precoated with silica gel 60 F254 (Merck KGaA, Darmstadt, Germany). Visualization was performed with UV light at a wavelength of 254 nm. Purifications were conducted with a flash column chromatography system (PuriFlash, Interchim, Montluçon, France) using stepwise gradients of petroleum ether (PE)/dichloromethane (DCM) or ethyl acetate (EtOAc) as the eluent. Melting points were measured with an SMP3 Melting Point instrument (STUART, Bibby Scientific Ltd., Roissy, France) with a precision of 1.5 °C. IR spectra were recorded with a Spectrum 100 Series FTIR spectrometer (PerkinElmer, Villebon S/Yvette, France). NMR spectra (^1^H and ^13^C) were acquired at 295 K using an AVANCE 300 MHz spectrometer (Bruker, Wissembourg, France) at 300 and 75.4 MHz. Coupling constant *J* was in Hz and chemical shifts were given in ppm. Mass (ESI, EI and field desorption (FD) were recorded with an LCP 1er XR spectrometer (WATERS, Guyancourt, France). Microwave experiments at atmospheric pressure were carried out in RotoSYNTH (0–1200 W) (Milestone Srl, Italy). Microwave reactions in sealed tubes (10 mL) were performed with an Initiator microwave synthesis instrument (0–400W) (Biotage, Uppsala, Sweden). The percentage of purity of all tested products was more than 95% (determined by HPLC analysis). ^1^H-NMR and ^13^C-NMR spectra of new compounds are available in Supplementary Materials (Section Figures S1–S12).

All details concerning the synthesis of cyanoenamines (**1a–d**) are described in preceding work [26,27,28]. **MPC-6827** was synthesized according to our previous methods [20].

#### 4.1.2. General Procedure for the Synthesis of *N*,*N*-dimethylacetimidamide Derivatives (**2a–d**)

A mixture of the appropriate cyanoenamine (3.0 mmol) and DMA-DMA (4 mL, 30 mmol) was heated under microwave irradiation (800 W). On completion, the reaction was cooled to room temperature and crude products were extracted 3 times with EtOAc (5 mL). The organic layers were washed with cold water, dried over Na_2_SO_4_, filtered and evaporated in vacuo. The crude product was purified by silica gel column chromatography using PE/DCM (100:0–0:100, *v*/*v*) as the eluent to give the desired products.

*(E)-N′-(2-Cyanobenzo[b]thiophen-3-yl)-N,N-dimethylacetimidamide* (**2a**): Orange powder (0.502 g, 72%) obtained from 3-aminobenzothiophene-2-carbonitrile (**1a**) after 5 min at 115 °C according to the general procedure; mp: 92–93 °C; IR (neat) ν_max_ (cm^−1^): 2199 (CN), 1591, 1557, 1476, 1456, 1426, 1414, 1397, 1365, 1346, 1318, 1188, 1059, 1024, 1016, 934, 887, 755, 737; ^1^H NMR (300 MHz, DMSO-*d*_6_): δ 7.98 (dd, 1H, *J*_1_ = 1 Hz, *J*_2_ = 8 Hz, *H*-7), 7.66 (dd, 1H, *J*_1_ = 1 Hz, *J*_2_ = 8 Hz, *H*-4), 7.58 (td, 1H, *J*_1_ = 1 Hz, *J*_2_ = 8 Hz, *H*-6), 7.45 (td, 1H, *J*_1_ = 1 Hz, *J*_2_ = 8 Hz, *H*-5), 3.34 (s, 3H, NC*H*_3_), 3.12 (s, 3H, NC*H*_3_), 1.97 (s, 3H, C*H*_3_); ^13^C NMR (75 MHz, DMSO-*d*_6_): δ 159.6, 156.8, 138.8, 133.9, 128.5, 125.1, 123.6, 123.3, 115.9, 97.0, 40.0 (2C), 15.9; HRMS calculated for C_13_H_14_N_3_S [M + H]^+^ 244.0908 found 244.0908.

*(E)-N′-(2-Cyanothieno[3,2-b]pyridin-3-yl)-N,N-dimethylacetimidamide* (**2b**): Orange oil (0.513 g, 74%) obtained from 3-aminothieno[3,2-*b*]pyridine-2-carbonitrile (**1b**) after 5 min at 115 °C according to the general procedure; IR (neat) ν_max_ (cm^−1^): 2210 (CN), 1576, 1413, 1391, 1369, 1348, 1191, 1062, 1022, 794, 784; ^1^H NMR (300 MHz, DMSO-*d*_6_): δ 8.73 (dd, 1H, *J*_1_ = 2 Hz, *J*_2_ = 5 Hz, *H*-5), 8.50 (dd, 1H, *J*_1_ = 2 Hz, *J*_2_ = 8 Hz, *H*-7), 7.56 (dd, 1H, *J*_1_ = 5 Hz, *J*_2_ = 8 Hz, *H*-6), 3.11 (s, 6H, N(C*H*_3_)_2_), 1.89 (s, 3H, C*H*_3_); ^13^C NMR (75 MHz, DMSO-*d*_6_): δ 160.3, 156.4, 148.2, 147.3, 133.8, 132.1, 122.1, 115.7, 91.5, 40.2 (2C), 17.3; HRMS calculated for C_12_H_13_N_4_S [M + H]^+^ 245.0861 found 245.0852.

*(E)-N′-(2-Cyanothieno[2,3-b]pyridin-3-yl)-N,N-dimethylacetimidamide* (**2c**): Grey powder (0.578 g, 83%) obtained from 3-aminothieno[2,3-*b*]pyridine-2-carbonitrile (**1c**) after 5 min at 115 °C according to the general procedure; mp: 136–137 °C; IR (neat) ν_max_ (cm^−1^): 2201 (CN), 1575, 1553, 1448, 1429, 1417, 1399, 1381, 1369, 1342, 1186, 1058, 1024, 934, 809, 755, 736; ^1^H NMR (300 MHz, DMSO-*d*_6_): δ 8.76 (dd, 1H, *J*_1_ = 2 Hz, *J*_2_ = 5 Hz, *H*-4), 8.08 (dd, 1H, *J*_1_ = 2 Hz, *J*_2_ = 8 Hz, *H*-6), 7.52 (dd, 1H, *J*_1_ = 5 Hz, *J*_2_ = 8 Hz, *H*-5), 3.12 (s, 6H, N(C*H*_3_)_2_), 2.01 (s, 3H, C*H*_3_); ^13^C NMR (75 MHz, DMSO-*d*_6_): δ 160.1, 159.4, 154.9, 150.9, 132.0, 127.8, 120.6, 115.5, 86.2, 39.8 (2C), 16.1; HRMS calculated for C_12_H_13_N_4_S [M + H]^+^ 245.0861 found 245.0853.

*(E)-N′-(6-Cyanothieno[2,3-b]pyrazin-7-yl)-N,N-dimethylacetimidamide* (**2d**): Orange powder (0.424 g, 61%) obtained from 7-aminothieno[2,3-*b*]pyrazine-6-carbonitrile (**1d**) after 2 min at 100 °C according to the general procedure; mp: 139–140 °C; IR (neat) ν_max_ (cm^−1^): 2209 (CN), 1575, 1478, 1431, 1413, 1393, 1377, 1365, 1341, 1334, 1180, 1081, 1072, 1041, 1024, 946, 865, 745; ^1^H NMR (300 MHz, DMSO-*d*_6_): δ 8.83 (d, 1H, *J* = 8 Hz, *H*-5), 8.79 (d, 1H, *J* = 8 Hz, *H*-6), 3.12 (s, 6H, N(C*H*_3_)_2_), 1.94 (s, 3H, C*H*_3_); ^13^C NMR (75 MHz, DMSO-*d*_6_): δ 160.9, 154.5, 154.3, 144.3, 143.2, 141.8, 115.1, 91.1, 39.9 (2C), 17.3; HRMS calculated for C_11_H_12_N_5_S [M + H]^+^ 246.0813 found 246.0815.

#### 4.1.3. General Procedure for the Synthesis of *N*-(4-methoxyphenyl)-2-methylthieno[3,2-d]pyrimidin-4-amines (**3a–d**)

A mixture of *N*,*N*-dimethylacetimidamide derivatives (**2a**–**d**) (1.0 mmol), *p*-anisidine (1.0 mmol) and aluminium chloride (1.0 mmol) in MeCN (4 mL)/AcOH (2 mL) was heated under microwave irradiation (400 W). On completion, the reaction was cooled to room temperature and water was added. The solid was filtered off, washed twice with water and dried. The crude solid was purified by silica gel column chromatography using PE/EtOAc (100:0–0:100, *v*/*v*) as the eluent to give the desired products.

*N-(4-Methoxyphenyl)-2-methylbenzo[4,5]thieno[3,2-d]pyrimidin-4-amine* (**3a**): Pale yellow powder (0.250 g, 78%) obtained from **2a** after 60 min at 160 °C according to the general procedure; mp: 263–264 °C; IR (neat) ν_max_ (cm^−1^): 1605, 1582, 1569, 1509, 1432, 1388, 1301, 1250, 1170, 1124, 1098, 1023, 894, 826, 774, 743; ^1^H NMR (300 MHz, DMSO-*d*_6_): δ 8.78 (s, 1H, N*H*), 8.22 (dd, 1H, *J*_1_ = 1 Hz, *J*_2_ = 7 Hz, *H*-9), 7.75 (dd, 1H, *J*_1_ = 1 Hz, *J*_2_ = 7 Hz, *H*-8), 7.68-7.58 (m, 4H, *H*-6, *H*-7 and *H*-ar), 7.04 (d, 2H, *J* = 9 Hz, *H*-ar), 3.82 (s, 3H, OC*H*_3_), 2.73 (s, 3H, C*H*_3_); ^13^C NMR (75 MHz, DMSO-*d*_6_): δ 161.3, 158.6, 157.0, 156.1, 140.3 (2C), 130.4 (2C), 125.6 (2C), 123.8 (2C), 114.1 (3C), 112.2, 55.3, 23.5; HRMS calculated for C_18_H_16_N_3_OS [M + H]^+^ 322.1014 found 322.1026.

*N-(4-Methoxyphenyl)-2-methylpyrido[2’,3’:4,5]thieno[3,2-d]pyrimidin-4-amine* (**3b**): Yellow powder (0.119 g, 37%) obtained from **2b** after 60 min at 160 °C according to the general procedure; mp: >300 °C; IR (neat) ν_max_ (cm^−1^): 1561, 1546, 1515, 1497, 1441, 1410, 1372, 1337, 1296, 1247, 1231, 1176, 1090, 1006, 813, 771, 742; ^1^H NMR (300 MHz, DMSO-*d*_6_): δ 9.62 (s, 1H, N*H*), 8.81 (dd, 1H, *J*_1_ = 2 Hz, *J*_2_ = 5 Hz, *H*-9), 8.63 (dd, 1H, *J*_1_ = 2 Hz, *J*_2_ = 8 Hz, *H*-7), 7.65-7.59 (m, 3H, *H*-8 and *H*-ar), 6.97 (d, 2H, *J* = 9 Hz, *H*-ar), 3.79 (s, 3H, OC*H*_3_), 2.58 (s, 3H, C*H*_3_); ^13^C NMR (75 MHz, DMSO-*d*_6_): δ 163.7, 161.6, 156.2, 155.5, 154.3, 151.5, 131.3 (2C), 127.4, 124.9, 120.8, 113.7 (3C), 110.9, 55.2, 25.6; HRMS calculated for C_17_H_15_N_4_OS [M + H]^+^ 323.0967 found 323.0960.

*N-(4-Methoxyphenyl)-2-methylpyrido[3’,2’:4,5]thieno[3,2-d]pyrimidin-4-amine* (**3c**): Yellow powder (0.087 g, 27%) obtained from **2c** after 60 min of irradiation at 160 °C according to the general procedure; mp: 221–222 °C; IR (neat) ν_max_ (cm^−1^): 1579, 1539, 1506, 1410, 1381, 1260, 1067, 982, 824, 748; ^1^H NMR (300 MHz, DMSO-*d*_6_): δ 9.65 (s, 1H, N*H*), 8.83 (dd, 1H, *J*_1_ = 2 Hz, *J*_2_ = 5 Hz, *H*-8), 8.63 (dd, 1H, *J*_1_ = 2 Hz, *J*_2_ = 8 Hz, *H*-6), 7.65-7.62 (m, 3H, *H*-7 and *H*-ar), 6.98 (d, 2H, *J* = 9 Hz, *H*-ar), 3.79 (s, 3H, OC*H*_3_), 2.60 (s, 3H, C*H*_3_); HRMS calculated for C_17_H_15_N_4_OS [M + H]^+^ 323.0967 found 323.0975.

*N-(4-Methoxyphenyl)-2-methylpyrazino[2’,3’:4,5]thieno[3,2-d]pyrimidin-4-amine* (**3d**): Yellow powder (0.191 g, 59%) obtained from **2d** after 60 min at 140 °C according to the general procedure; mp: 266–267 °C; IR (neat) ν_max_(cm^−1^): 1551, 1515, 1500, 1436, 1412, 1372, 1333, 1299, 1238, 1176, 1096, 1088, 1032, 1002, 861, 839, 765, 724; ^1^H NMR (300 MHz, DMSO-*d*_6_): δ 9.76 (s, 1H, N*H*), 8.93 (d, 1H, *J* = 8 Hz, *H*-7), 8.92 (d, 1H, *J* = 8 Hz, *H*-8), 7.65 (d, 2H, *J* = 9 Hz, *H*-ar), 6.99 (d, 2H, *J* = 9 Hz, *H*-ar), 3.79 (s, 3H, OC*H*_3_), 2.54 (s, 3H, C*H*_3_); ^13^C NMR (75 MHz, DMSO-*d*_6_): δ 164.4, 156.6, 156.4, 155.7, 152.7, 145.0, 143.5, 143.0, 131.1, 125.0, 113.8 (3C), 113.4, 55.2, 25.6; HRMS calculated for C_16_H_14_N_5_OS [M + H]^+^ 324.0919 found 324.0917.

#### 4.1.4. General Procedure for the Synthesis of *N*-(4-methoxyphenyl)-*N*,2-dimethylthieno[3,2-*d*]pyrimidin-4-amines (**4a–d**)

A suspension of *N*-(4-methoxyphenyl)-2-methylthieno[3,2-*d*]pyrimidin-4-amine (4.0 mmol) in DMF (5 mL) was cooled to 0 °C. Then, NaH (60% in mineral oil, 8.0 mmol) and methyl iodide (96 mmol) were added. The reaction mixture was stirred at 0 °C for 1 h and warmed to room temperature. The reaction was quenched by adding water. EtOAc was added and the organic phase was washed twice with H_2_O and brine, dried over MgSO_4_, filtered and evaporated under vacuum. The crude solid was purified by silica gel column chromatography using PE/EtOAc (100:0–0:100, *v*/*v*) as the eluent to give the desired product.

*N-(4-Methoxyphenyl)-N,2-dimethylbenzo[4,5]thieno[3,2-d]pyrimidin-4-amine* (**4a**): White powder (0.090 g, 86%) obtained from **3a** according to the general procedure; mp: 147–148 °C; IR (neat) ν_max_ (cm^−1^): 1539, 1525, 1508, 1456, 1440, 1390, 1369, 1346, 1247, 1165, 1145, 1093, 1032, 1008, 838, 754, 734; ^1^H NMR (300 MHz, DMSO-*d*_6_): δ 8.26 (dd, 1H, *J*_1_ = 1 Hz, *J*_2_ = 7 Hz, *H*-9), 7.87 (dd, 1H, *J*_1_ = 1 Hz, *J*_2_ = 7 Hz, *H*-8), 7.57-7.43 (m, 4H, *H*-6, *H*-7 and *H*-ar), 7.08 (d, 2H, *J* = 9 Hz, *H*-ar), 3.86 (s, 3H, OC*H*_3_), 3.52 (s, 3H, C*H*_3_), 2.65 (s, 3H, C*H*_3_); ^13^C NMR (75 MHz, DMSO-*d*_6_): δ 163.2, 159.2, 157.2, 156.7, 140.5, 135.4, 132.8, 130.6, 129.3 (2C), 124.8, 123.0, 122.6, 114.6 (2C), 111.8, 55.4, 39.6, 25.5; HRMS calculated for C_19_H_18_N_3_OS [M + H]^+^ 336.1171 found 336.1163.

*N-(4-Methoxyphenyl)-N,2-dimethylpyrido[2’,3’:4,5]thieno[3,2-d]pyrimidin-4-amine* (**4b**): Yellow powder (0.094 g, 90%) obtained from **3b** according to the general procedure; mp: >300 °C; IR (neat) ν_max_ (cm^−1^): 2921, 1383, 1248, 1077, 1033, 860; ^1^H NMR (300 MHz, DMSO-*d*_6_): δ 8.76 (dd, 1H, *J*_1_ = 2 Hz, *J*_2_ = 5 Hz, *H*-8), 8.39 (dd, 1H, *J*_1_ = 2 Hz, *J*_2_ = 8 Hz, *H*-6), 7.53 (dd, 1H, *J*_1_ = 5 Hz, *J*_2_ = 8 Hz, *H*-7), 7.45 (d, 2H, *J* = 9 Hz, *H*-ar), 7.09 (d, 2H, *J* = 9 Hz, *H*-ar), 3.86 (s, 3H, OC*H*_3_), 3.53 (s, 3H, C*H*_3_), 2.67 (s, 3H, C*H*_3_); HRMS calculated for C_18_H_17_N_4_OS [M + H]^+^ 337.1123 found 337.1133.

*N-(4-Methoxyphenyl)-N,2-dimethylpyrido[3’,2’:4,5]thieno[3,2-d]pyrimidin-4-amine* (**4c**): White powder (0.087 g, 83%) obtained from **3c** according to the general procedure; mp: 161–162 °C; IR (neat) ν_max_ (cm^−1^): 1539, 1510, 1443, 1415, 1395, 1375, 1349, 1303, 1252, 1169, 1097, 1029, 979, 873, 835, 772, 748; ^1^H NMR (300 MHz, DMSO-*d*_6_): δ 8.70 (dd, 1H, *J*_1_ = 2 Hz, *J*_2_ = 5 Hz, *H*-9), 8.59 (dd, 1H, *J*_1_ = 2 Hz, *J*_2_ = 8 Hz, *H*-7), 7.54 (dd, 1H, *J*_1_ = 5 Hz, *J*_2_ = 8 Hz, *H*-8), 7.45 (d, 2H, *J* = 9 Hz, *H*-ar), 7.09 (d, 2H, *J* = 9 Hz, *H*-ar), 3.88 (s, 3H, OC*H*_3_), 3.54 (s, 3H, C*H*_3_), 2.65 (s, 3H, C*H*_3_); ^13^C NMR (75 MHz, DMSO-*d*_6_): δ 163.7, 161.9, 159.5, 157.1, 154.6, 151.5, 135.1, 131.4, 130.9 (2C), 127.0, 120.6, 115.0 (2C), 111.0, 55.6, 40.7, 25.8; HRMS calculated for C_18_H_17_N_4_OS [M + H]^+^ 337.1123 found 337.1119.

*N-(4-Methoxyphenyl)-N,2-methylpyrazino[2’,3’:4,5]thieno[3,2-d]pyrimidin-4-amine* (**4d**): Brown powder (0.077 g, 74%) obtained from **3d** according to the general procedure; mp: 238–239 °C; IR (neat) ν_max_ (cm^−1^): 1531, 1505, 1480, 1458, 1439, 1394, 1355, 1344, 1244, 1179, 1173, 1159, 1108, 1090, 1031, 1011, 864, 834, 800, 764; ^1^H NMR (300 MHz, DMSO-*d*_6_): δ 8.83 (d, 1H, *J* = 8 Hz, *H*-7), 8.81 (d, 1H, *J* = 8 Hz, *H*-8), 7.47 (d, 2H, *J* = 9 Hz, *H*-ar), 7.10 (d, 2H, *J* = 9 Hz, *H*-ar), 3.87 (s, 3H, OC*H*_3_), 3.54 (s, 3H, C*H*_3_), 2.67 (s, 3H, C*H*_3_); ^13^C NMR (75 MHz, DMSO-*d*_6_): δ 164.2, 159.6, 157.2, 156.7, 153.0, 145.0, 142.8 (2C), 134.7, 131.0 (2C), 114.8 (2C), 113.2, 55.5, 39.1, 25.7; HRMS calculated for C_17_H_16_N_5_OS [M + H]^+^ 338.1076 found 338.1073.

### 4.2. Antiproliferative Activity

The antiproliferative activity was estimated according to methods previously described [21].

Human HT-29 and Caco-2 colorectal adenocarcinoma cells were purchased from American Type Culture Collection (Manassas, VA, USA). HT-29 and Caco-2 colorectal cancer cell lines possess different p53 mutations [29]. The cells were grown in Dulbecco’s modified Eagle’s medium supplemented with 20% fetal bovine serum (Invitrogen, France), 2 mM L-glutamine (Invitrogen,) and penicillin (10 U/mL)/streptomycin (10 µg/mL) at 37 °C with 5% CO_2_ and 90% relative humidity. Dimethyl sulfoxide (DMSO) was purchased from Sigma-Aldrich, France.

MTT proliferation assay: Caco-2 and HT-29 cells were seeded at a density of 8000 cells/well in 96-well microplates. Cells were treated after 24 h and allowed to proliferate with or without molecules, for 4 days. Molecules were tested at indicated concentrations after extemporaneous dilution in a medium of a starting solution (100 mmol/L in DMSO). The final concentration of DMSO in culture medium was maintained at 0.1%. Molecule **3a** was not tested because of its poor solubility in the conditions used. The MTT test was carried out daily after treatment (1, 24, 48 or 72 h).

For the tested molecules, data of statistical analysis were presented as mean ± SD from at least three independent experiments. At least six different replicates were conducted for each compound. A statistical analysis was performed with non-parametric test (Mann–Whitney U test between two groups) using the SigmaStat software. Differences were considered to be statistically significant at a *P* value < 0.05. No effect of the solvent was observed. The results obtained for the treated cells were compared with those obtained with cells cultured in the presence of DMSO, which represented 100% of proliferation (control).

## Figures and Tables

**Table 1:** Experimental conditions for the synthesis of compounds **2**, **3** and **4**.

Compound 2	Temp ^1^ (°C)	Time ^2^ (min)	Yield ^3^ (%)	Compound 3	Temp ^1^ (°C)	Yield ^3^ (%)	Compound 4	Yield ^3^ (%) ^1^
**a**	115	5	72	**a**	160	78	**a**	86
**b**	115	5	74	**b**	160	37	**b**	90
**c**	115	5	83	**c**	160	27	**c**	83
**d**	100	2	61	**d**	140	59	**d**	74

^1^ Temperature; ^2^ reaction time; ^3^ isolated yields.

## Supplementary Materials

The following materials are available online at https://www.mdpi.com/1424-8247/13/9/202/s1, ^1^H-NMR and ^13^C-NMR spectra of new compounds.
