# Supplementary material for: Microwave-Assisted Synthesis of Potential Bioactive Benzo-, Pyrido- or Pyrazino-thieno[3,2-d]pyrimidin-4-amine Analogs of MPC-6827
Source: Pharmaceuticals (Basel). 2020 Aug 19;13(9):202. doi: 10.3390/ph13090202 (PMC7558077; doi:10.3390/ph13090202)
Supplement: Supplementary file 1 [file pharmaceuticals-13-00202-s001.pdf]

pharmaceuticals

MDPI

Supplementary Materials

Communication

Synthesis of Thieno[3,2-*d*]pyrimidine Analogs of MPC-6827 and Antiproliferative Activity on HT-29 and Caco-2 Colorectal Cancer Cells

Yvonnick Loidreau [1], Marie-Renée Nourrisson [2], Corinne Fruit [1], Cécile Corbière [3], Pascal Marchand [2,*] and Thierry Besson [1,*]

[1] Normandie Univ, UNIROUEN, INSA Rouen, CNRS, COBRA UMR 6014, 76000 Rouen, France; yvonnick.loidreau@gmail.com (Y.L.); corinne.fruit@univ-rouen.fr (C.F.)

[2] Université de Nantes, Cibles et médicaments des infections et du cancer, IICiMed, EA 1155, F-44000 Nantes, France; marie-renee.nourrisson@univ-nantes.fr (M.R.N.)

[3] Normandie Univ, UNIROUEN, ABTE, 76000 Rouen, France; cecile.corbiere@univ-rouen.fr (C.C.)

* Correspondence: pascal.marchand@univ-nantes.fr; Tel.: +33 253-009-155 (P.M.); thierry.besson@univ-rouen.fr; Tel.: +33 235-522-904 (T.B.)

1. 1H- & 13C-NMR Spectra for Compounds 2a-2d, 3a-3d and 4a-4d

Figure S1. 1H NMR at 300 MHz and 13C NMR at 75.4 MHz spectra, DMSO-d6, for compound 2a.

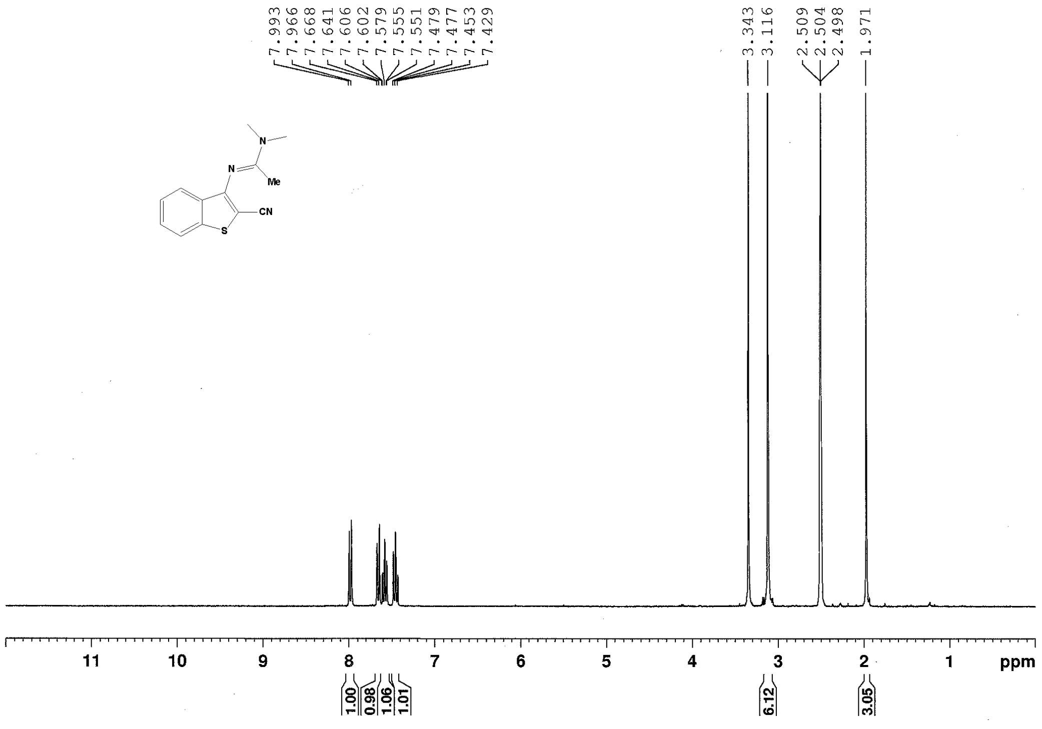

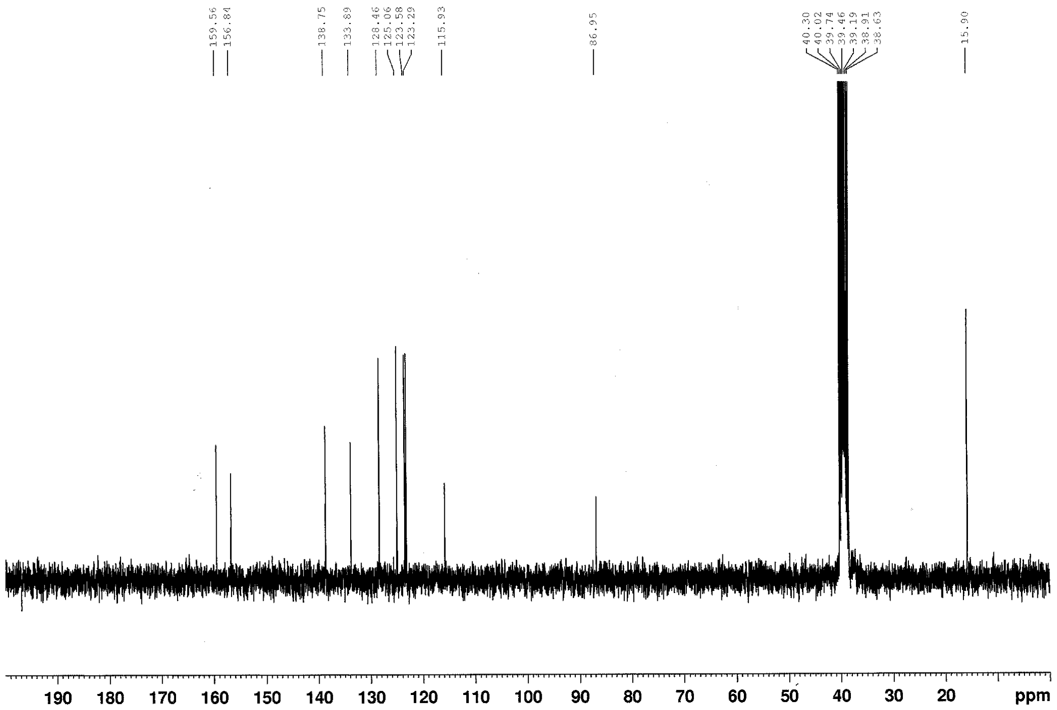

Figure S2. 1H NMR at 300 MHz and 13C NMR at 75.4 MHz spectra, DMSO-d6, for compound 2b.

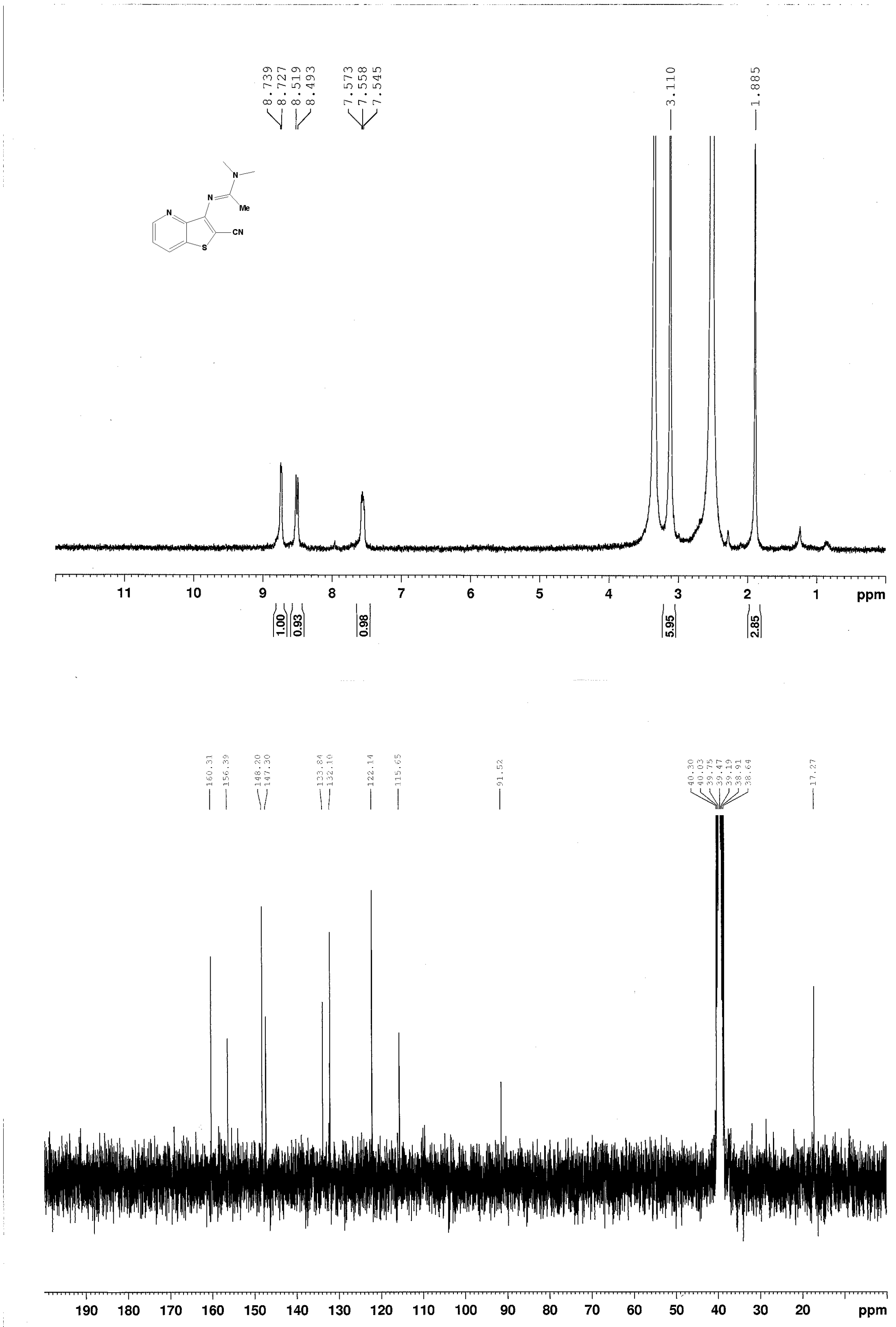

Figure S3. 1H NMR at 300 MHz and 13C NMR at 75.4 MHz spectra, DMSO-d6, for compound 2c

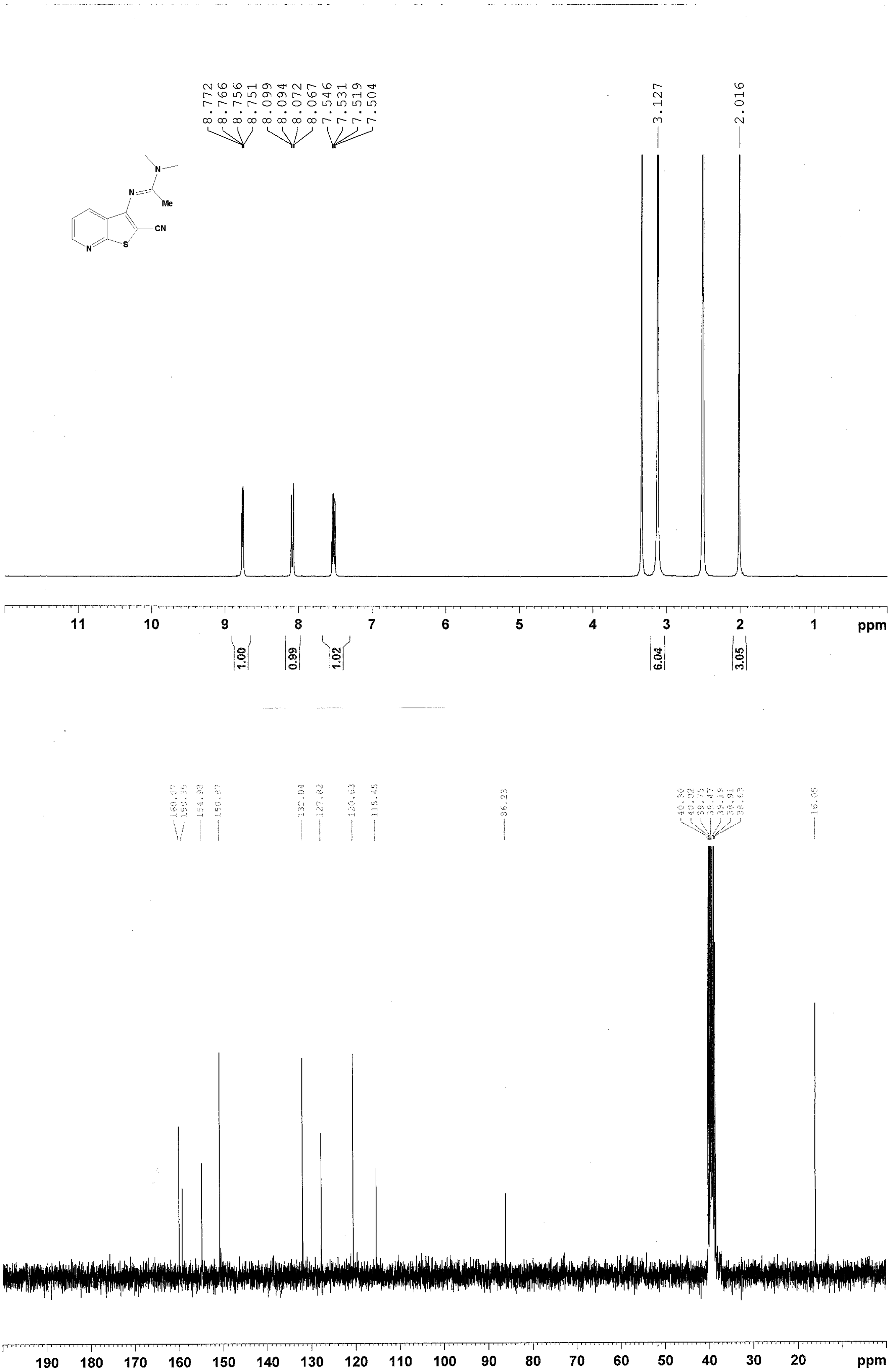

Figure S4. 1H NMR at 300 MHz and 13C NMR at 75.4 MHz spectra, DMSO-d6, for compound 2d

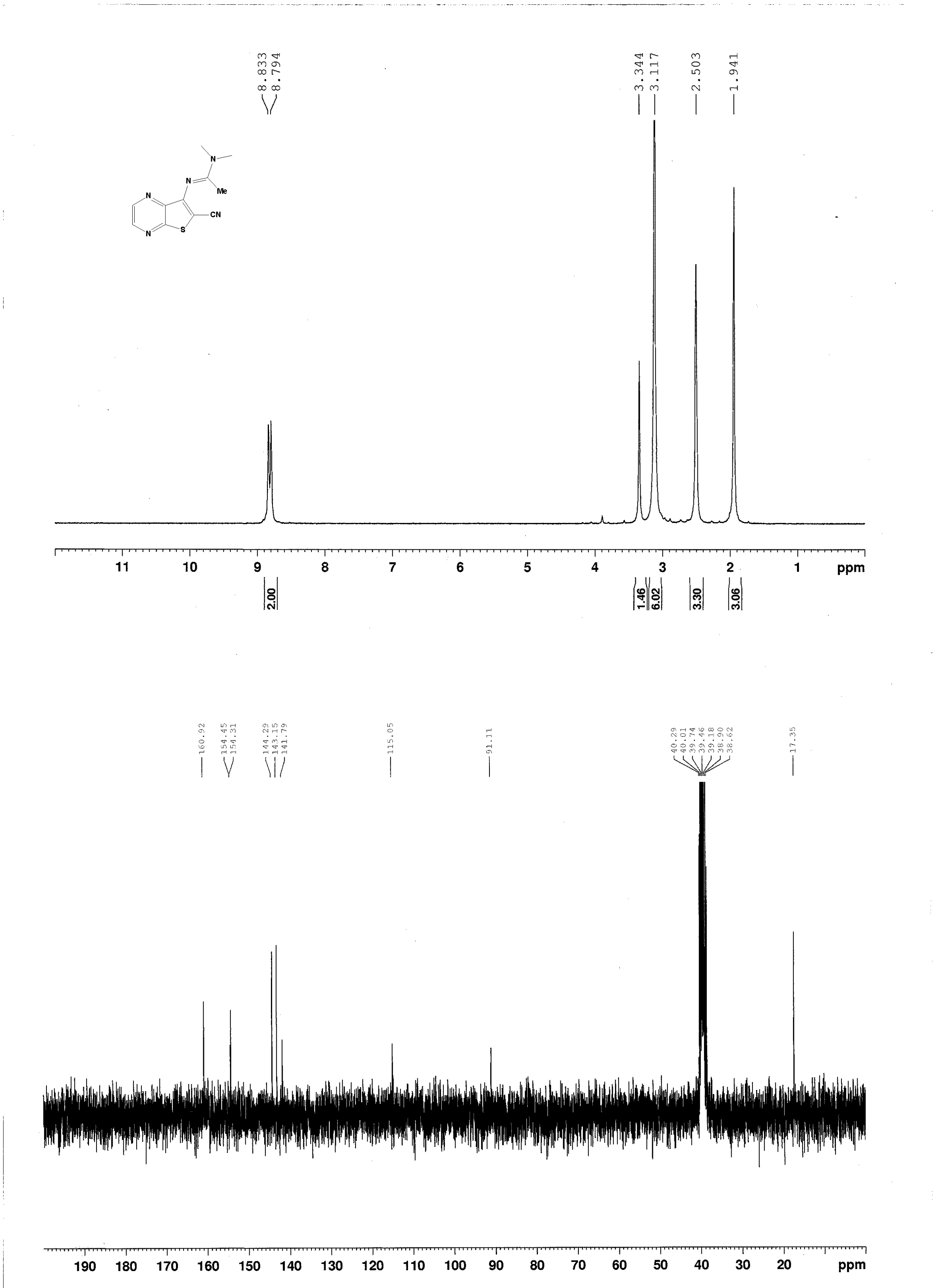

Figure S5. 1H NMR at 300 MHz and 13C NMR at 75.4 MHz spectra, DMSO-d6, for compound 3a

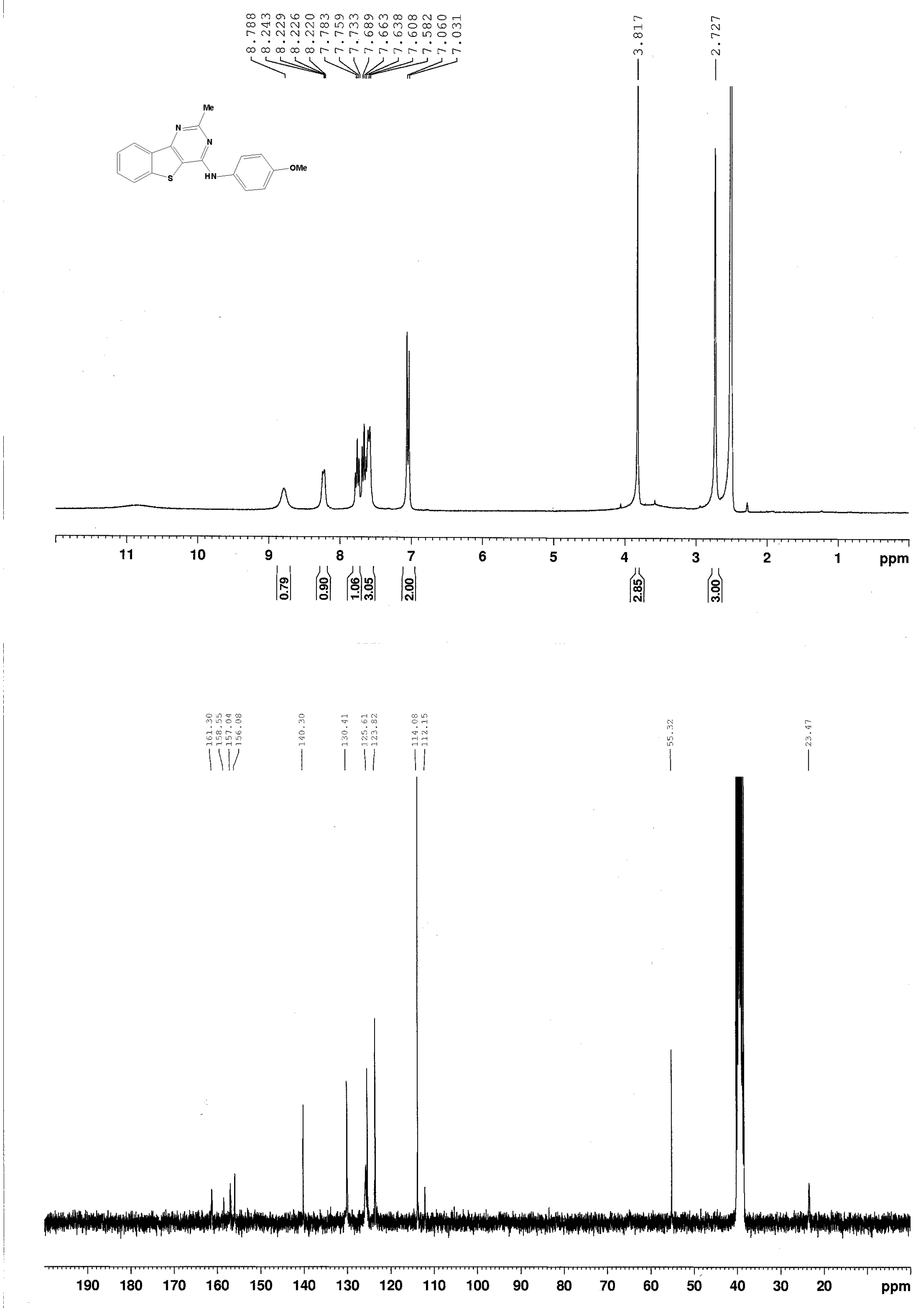

Figure S6. 1H NMR at 300 MHz and 13C NMR at 75.4 MHz spectra, DMSO-d6, for compound 3b

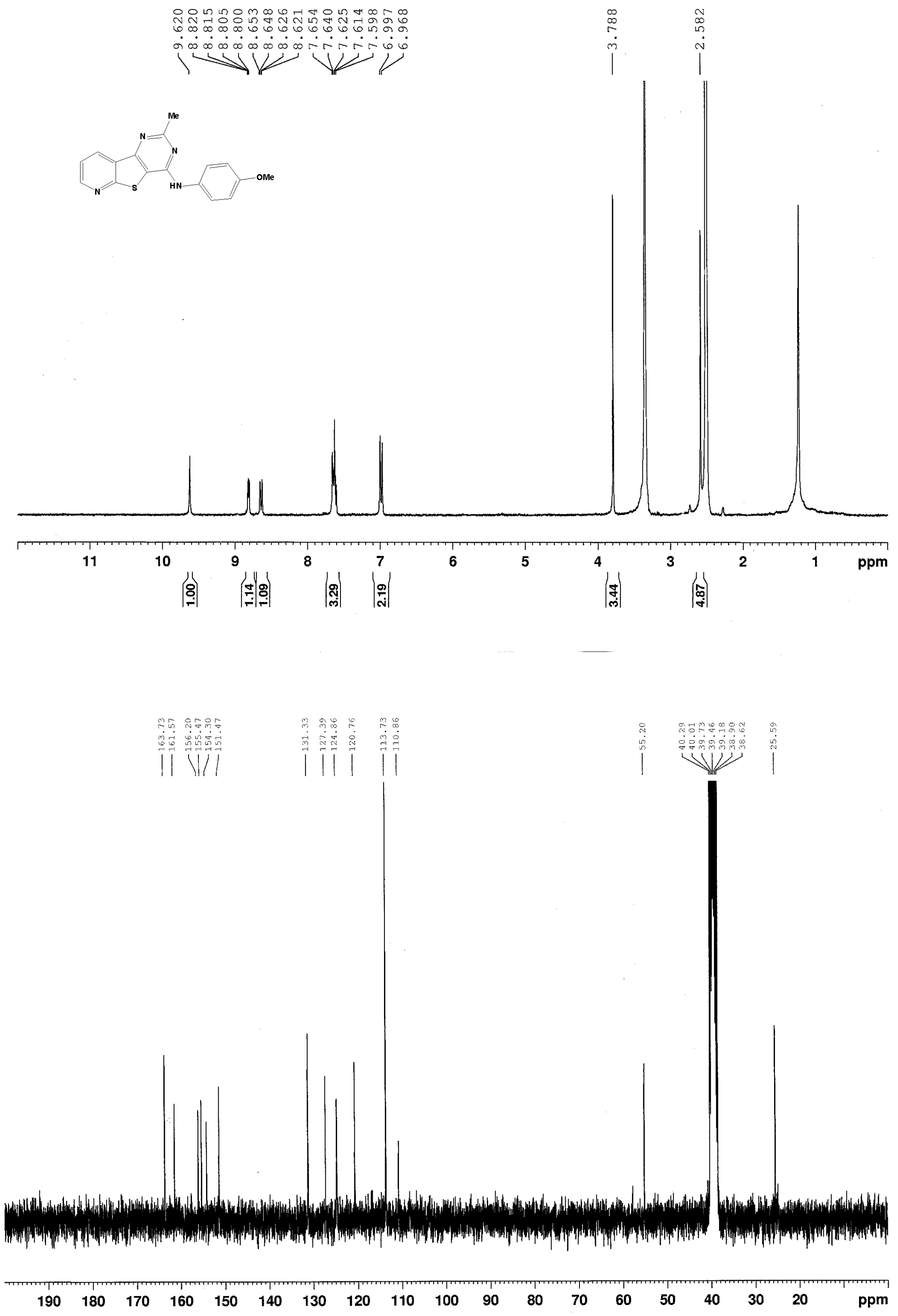

Figure S7. 1H NMR at 300 MHz and 13C NMR at 75.4 MHz spectra, DMSO-d6, for compound 3c

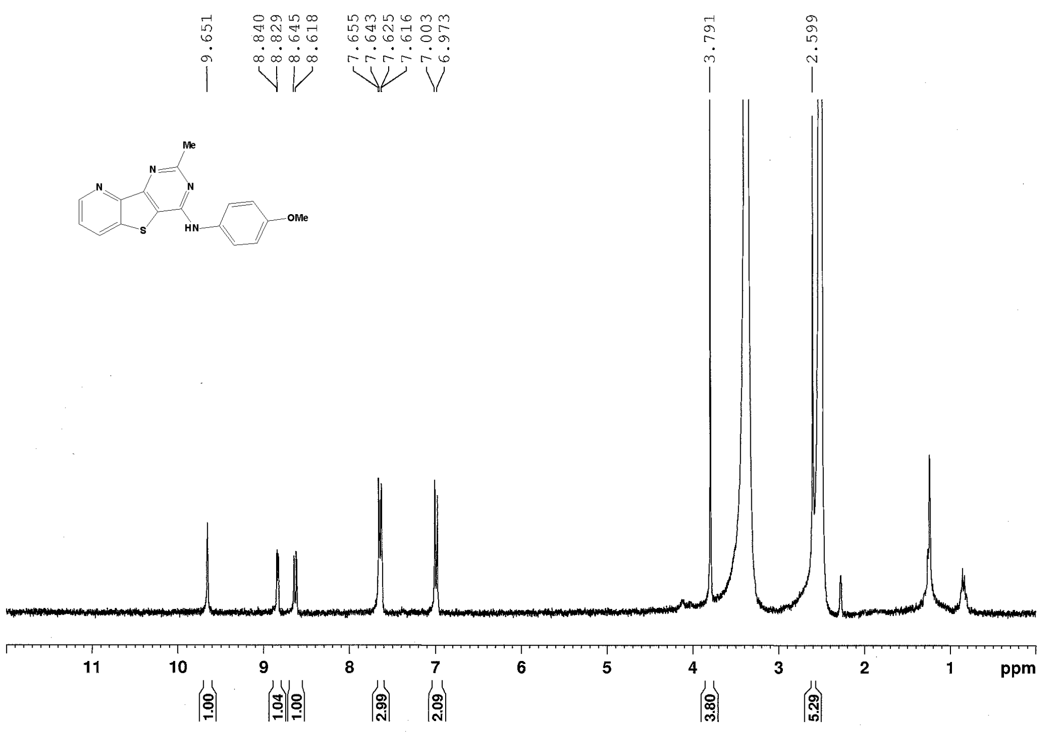

Figure S8. 1H NMR at 300 MHz and 13C NMR at 75.4 MHz spectra, DMSO-d6, for compound 3d

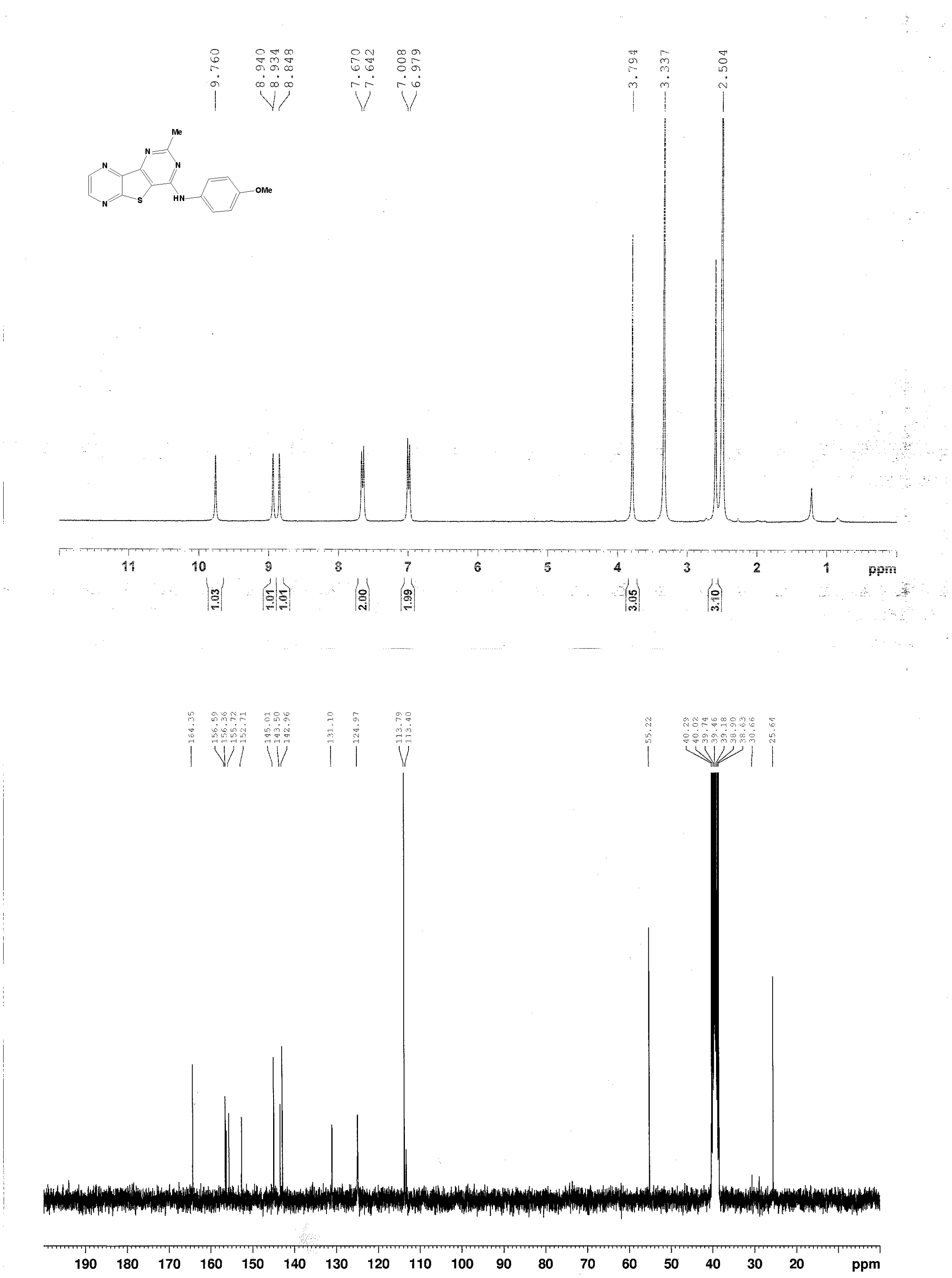

Figure S9. 1H NMR at 300 MHz and 13C NMR at 75.4 MHz spectra, DMSO-d6, for compound 4a

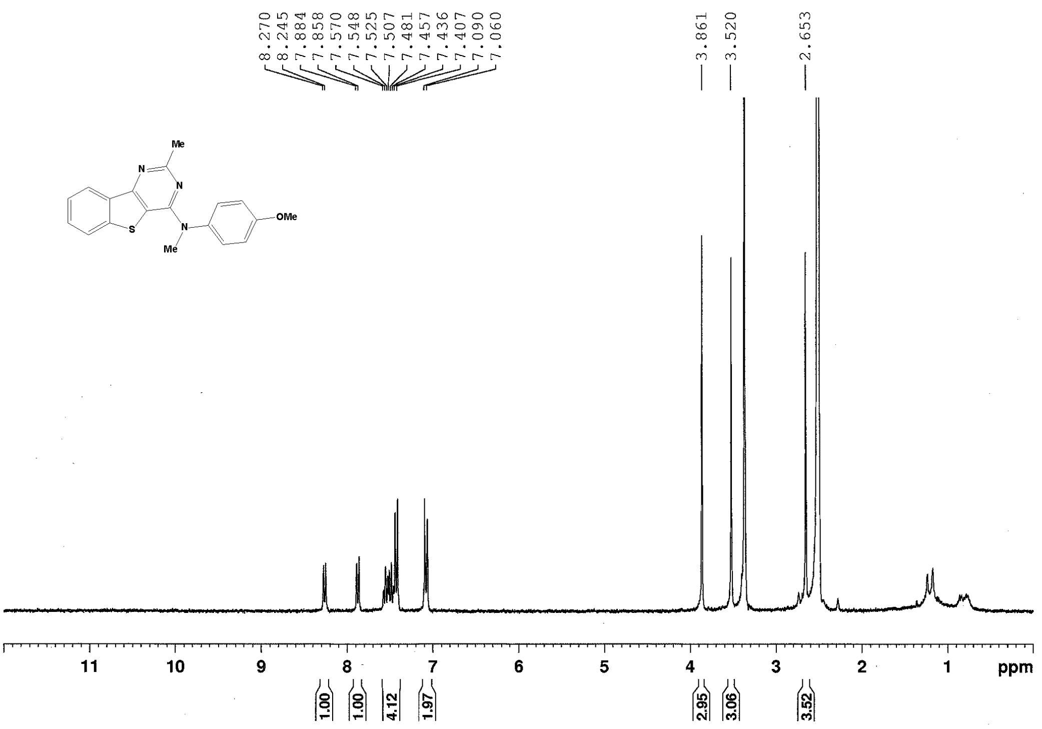

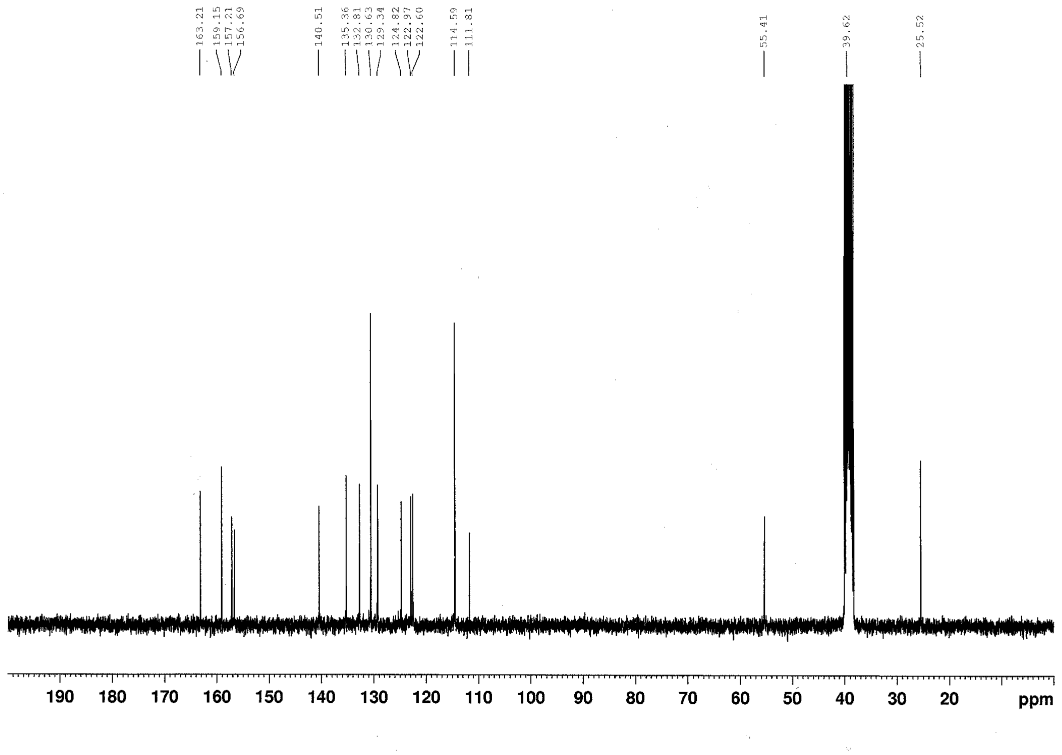

Figure S10. 1H NMR at 300 MHz and 13C NMR at 75.4 MHz spectra, DMSO-d6, for compound 4b

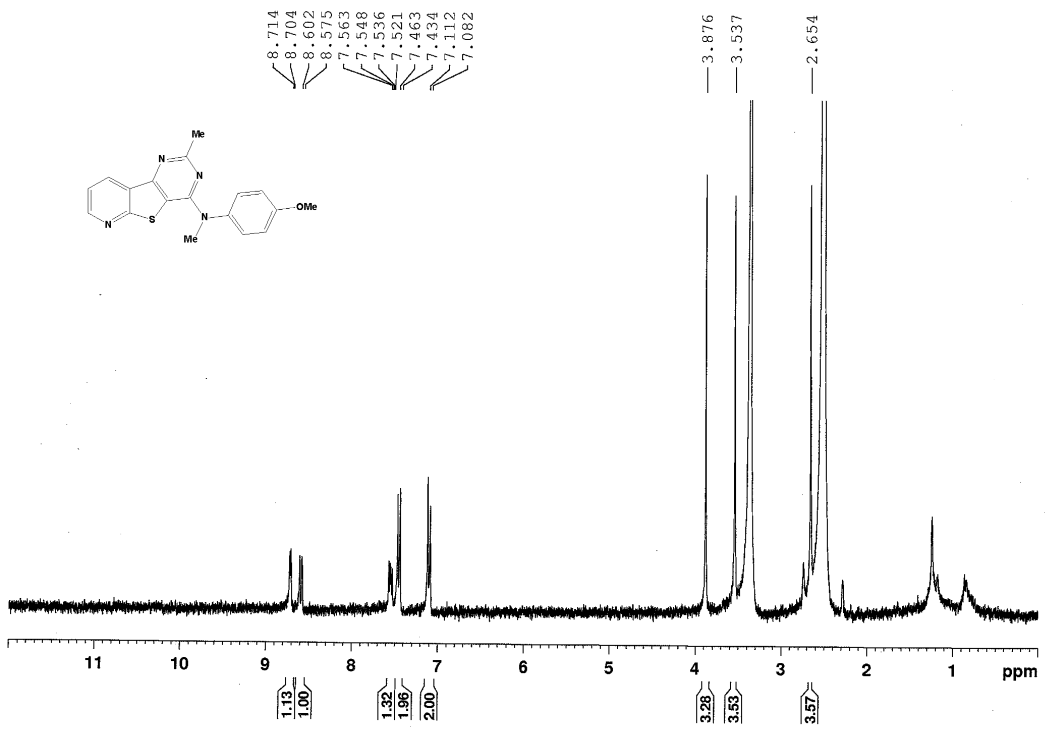

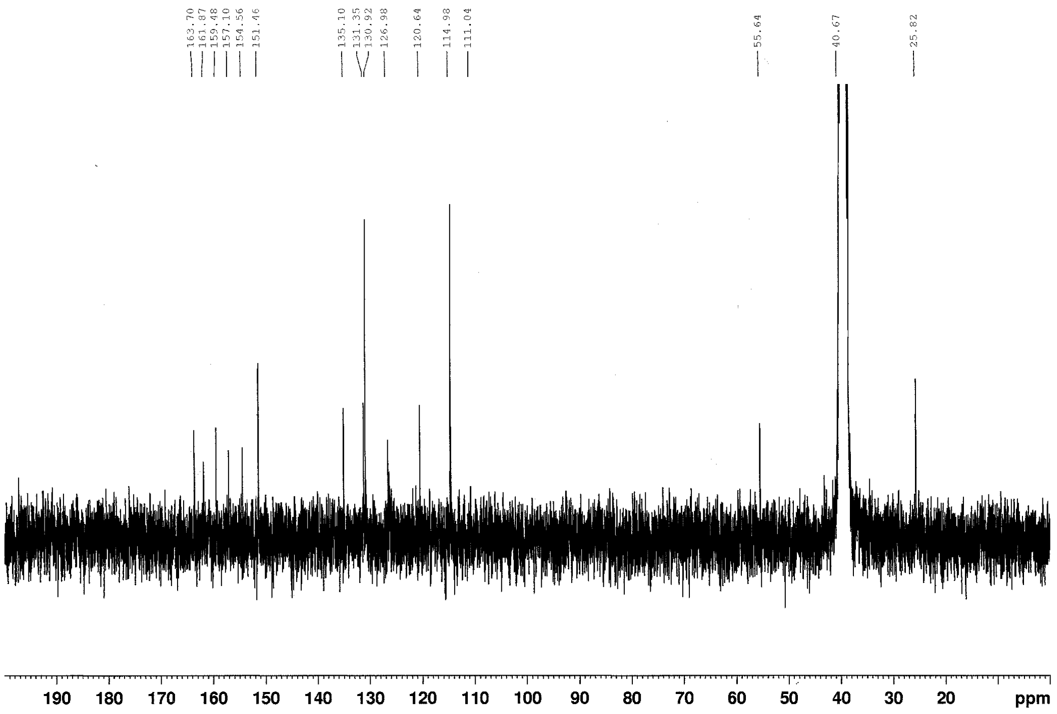

Figure S11. 1H NMR at 300 MHz and 13C NMR at 75.4 MHz spectra, DMSO-d6, for compound 4c

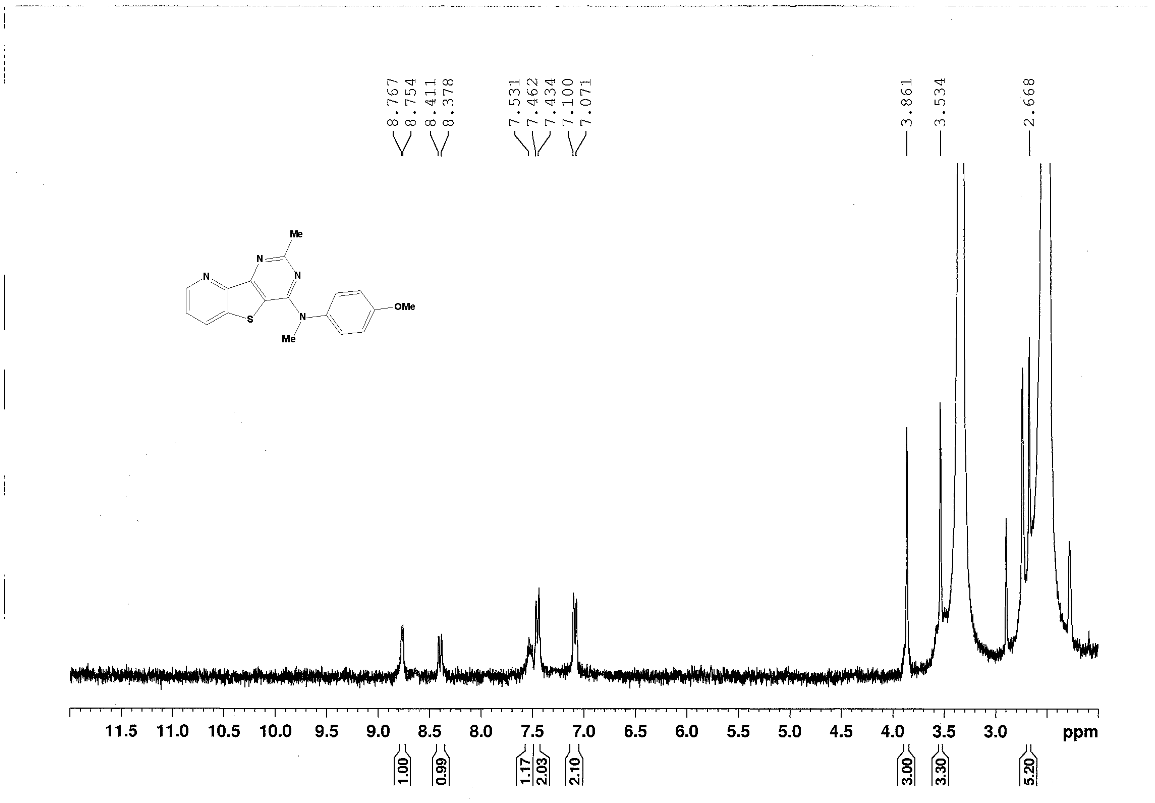

Figure S12. 1H NMR at 300 MHz and 13C NMR at 75.4 MHz spectra, DMSO-d6, for compound 4d

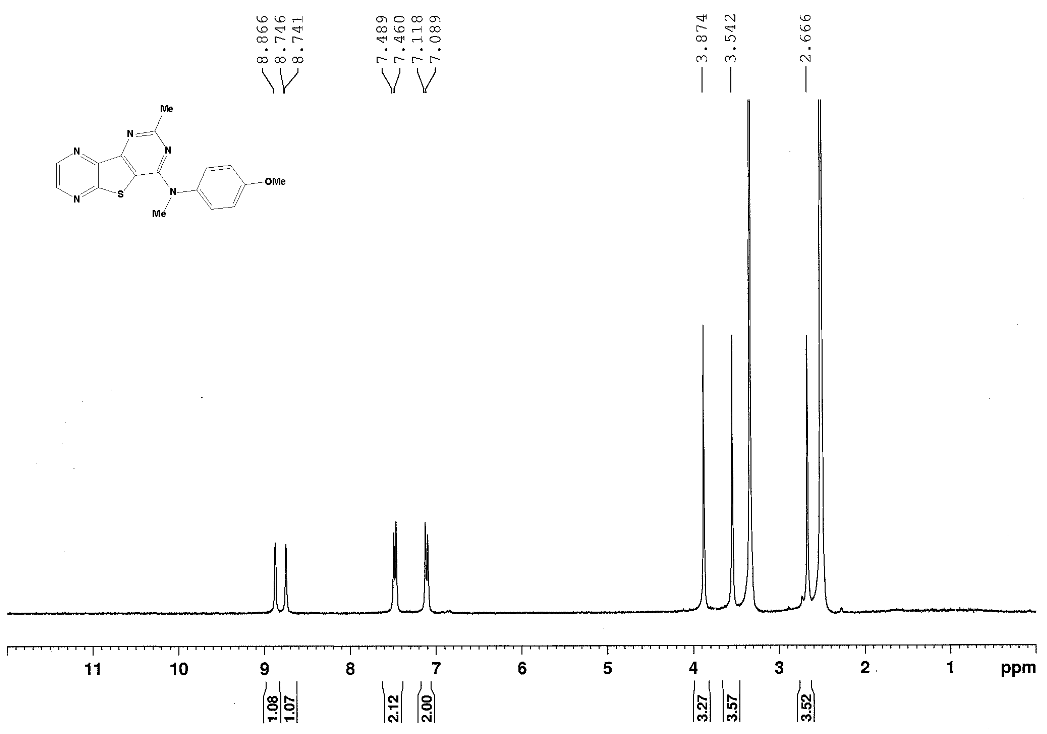

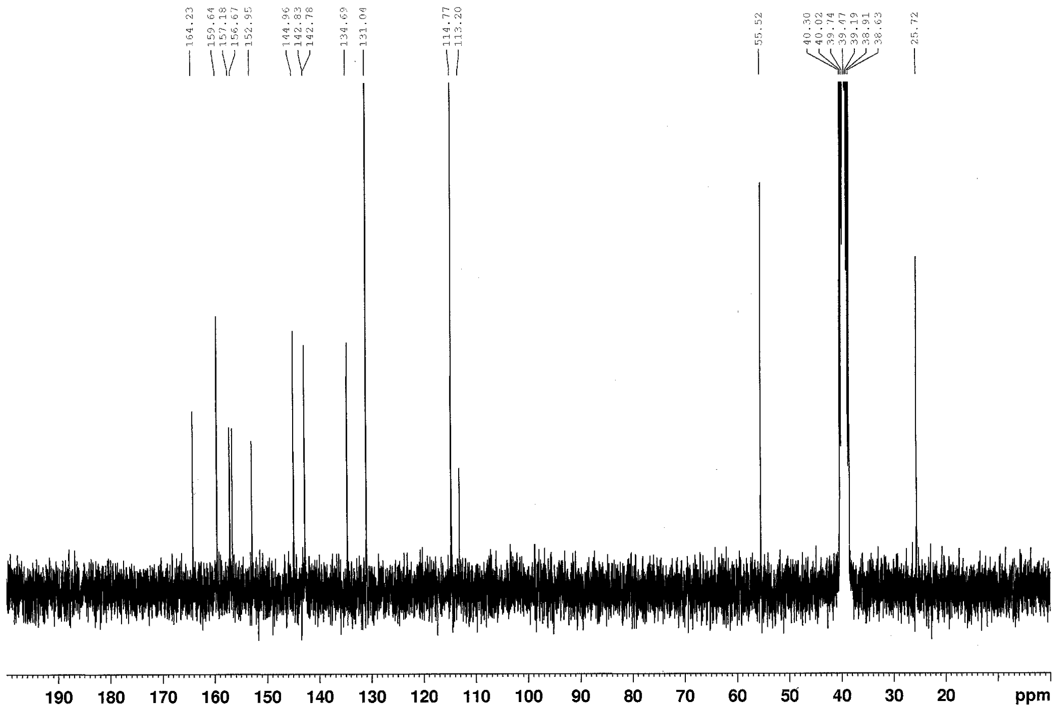

© 2020 by the authors. Submitted for possible open access publication under the terms and conditions of the Creative Commons Attribution (CC BY) license (http://creativecommons.org/licenses/by/4.0/).
